# Multiple hydride reduction pathways in isoflavonoids

**DOI:** 10.1186/1860-5397-2-16

**Published:** 2006-08-25

**Authors:** Auli K Salakka, Tuija H Jokela, Kristiina Wähälä

**Affiliations:** 1 Department of Chemistry, Laboratory of Organic Chemistry, P.O.Box 55, FIN-00014 University of Helsinki, Finland

## Abstract

**Background:**

Isoflavonoids are of interest owing to their appearance in metabolic pathways of isoflavones, and their estrogenic and other physiological properties, making them promising lead compounds for drug design.

**Results:**

The reduction of isoflavones by various hydride reagents occurs by a 1,4-pathway in contrast to ordinary β-alkoxy-α,β-unsaturated ketones. Isoflavan-4-ones, *cis*- and *trans*-isoflavan-4-ols, α-methyldeoxybenzoins or 1,2-diphenylprop-2-en-1-ols are obtained depending on the hydride reagent, mostly in good yields. The stereoselective reduction of isoflavan-4-ones is also discussed.

**Conclusion:**

The work described in this paper shows that most structural types of reduced isoflavonoids are now reliably available in satisfactory or good yields by hydride reductions to be used as authentic reference compounds in analytical and biological studies.

## Background

The reduction of isoflavones **1** has been actively studied during the last twenty years, owing to the range of interesting biological effects [1–3] – estrogenic activity, promise in cancer, osteoporosis, and coronary heart disease prevention – shown by the isoflavones themselves and their reduced metabolites. There are some reports [4–8] of total syntheses of reduced isoflavonoid structures from commercially available starting materials but overall yields in these multistep procedures tend to be low, and free hydroxy groups are not compatible. Another strategy involves the hydrogenation of isoflavones using a palladium or platinum catalyst but mixtures of reduction products are often formed [9–10]. Certain hydride reagents have been tested, as discussed below, for the reduction of simple or protected isoflavones but many of the early results are contradictory or rely on indeterminate product characterization.

**Figure 1:**
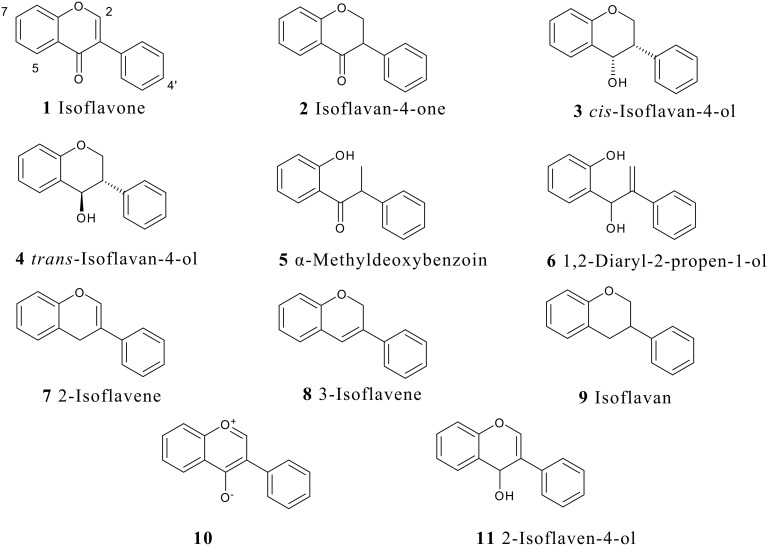
Isoflavonoid subclasses.

The reduction of simple (nonflavonoid) β-alkoxy-α,β-unsaturated ketones by hydride reagents (NaBH_4_, LiAlH_4_, DIBAH) occurs normally by 1,2-attack, this being a key step in the well known "carbonyl transposition" of 1,3-diketone enol ethers into enones (R^3^O-CR=CR^1^-COR^2^ → R-CO-CR^1^=CHR^2^). [11–15] Prior to our work there were no reports on the reduction of isoflavones containing free hydroxy groups which nevertheless are common, usually at one or several of the C-5, C-7 and C-4' sites, in the naturally occurring isoflavonoids. It is to be expected that the presence of the phenolic hydroxyls, or the derived phenolate anions, will alter the reactivity pattern of the parent isoflavone system, besides perhaps decreasing the overall reactivity due to solubility reasons. For example, any tendency of hydride attack at the C-2 will be opposed by electron feeding from the 4'-OH group while an OH group at C-5 or C-7 will discourage attack at C-2 and C-4. Thus there was ample room for the development of reliable methods for the synthesis of hydroxy-substituted isoflavone metabolites, and for clarification of the course of reduction of isoflavones with various hydride reducing agents. We present here experimental details of our own results in this field together with a thorough survey of the literature. The discussion is based on the types of reduced isoflavonoid structures formed (Figure 1), i.e., isoflavanones **2**, *cis*-isoflavan-4-ols **3**, *trans*-isoflavan-4-ols **4**, the ring opened α-methyldeoxybenzoins **5** and 1,2-diaryl-2-propen-1-ols **6**, and isoflavenes **7** and **8** (hydride reductions do not lead to isoflavans **9**, which however are obtained by catalytic hydrogenation [10,16,20]). Incidentally, it is appropriate to point out that flavones do not undergo similar reductive metabolism in mammals as described above for isoflavones. We will nevertheless present at a later date certain findings on the hydride reduction pathways in flavones.

## Results and Discussion

### Isoflavanones (2)

Isoflavanones result from the 1,4-reduction of isoflavones. The resonance contributor **10** will encourage this mode of attack. DIBAH, normally a preferential 1,2-reducer, reacts with methoxy-, benzyloxy-, MOMO- and MEMO-substituted isoflavones to give the isoflavanones in 40–93% yield [16,21–23] (Table 1). Our own work has shown that even *unprotected* hydroxy-substituted isoflavones are reduced by a large excess of DIBAH in 50–70% yield (Table 1). The simple borohydride reagents reduce isoflavones to the isoflavanols (see below) but the Selectrides^®^ give 60–88% yields of isoflavanones in the absence of hydroxy substituents according to our results (Table 1). There is no significant difference between the K- and L-Selectride^®^. In the literature, there is an isolated report of the reduction of a MOM substituted isoflavone by L-Selectride^®^ in 40% yield (Table 2) [16]. Methoxy substituted isoflavones have also been reduced by sodium hydrogen telluride to give isoflavan-4-ones in 61–71% yields [24].

**Table 1:** Reduction of isoflavones to isoflavanones.^a^

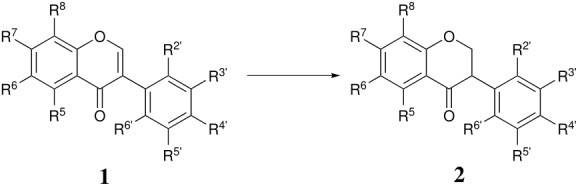										
**a** all R groups = H										
**b** R^7^ = OMe, other R groups = H										
**c** R^4'^ = R^7^ = OMe, other R groups = H										
**d** R^7^ = OH, other R groups = H										
**e** R^4'^ = OMe, R^7^ = OH, other R groups = H										
**f** R^4'^ = OMe, R^5^ = R^7^ = OH, other R groups = H										

R^2'^	R^3'^	R^4'^	R^5'^	R^6'^	R^5^	R^6^	R^7^	R^8^	reductant, eqs	yield %

									DIB^b^, 4	72
									SEL^c^, 4	96
							OH		DIB, 10	68
									SEL, 8	0
							OMe		DIB, 2.5	90^d,e^
									DIB, 6	57
									SEL, 4	84
					Me		OMe	OMe	DIB, 2.5	75^e^
					OMe	OMe	OMe		DIB, 2.5	89^e^
		OMe					OMe	OMe	NaHTe, 2	68^f^
		OH					OH		DIB, 25	70
									SEL, 8	0
		OMe					OH		DIB, 25	60
		OMe					OMe		DIB, 2.5	87^e^
									DIB, 7	54
									NaHTe, 2	61^f^
									SEL, 4	82
		OMOM					OMOM		DIB, 4.7	93^g^
		OH			OH		OH		DIB, 25	50
									SEL, 9	0
		OMe			OH		OH		DIB, 25	57
	OMe	OMe				OMe	OMe		NaHTe, 2	71^f^
OMOM		OMOM					OMOM		DIB, 4.7	87^g,h^
									SEL, 2	40^g^
OMe	OMe	OBn					OBn		DIB, 2.5	40^i^
OBn	OMe	OMe					OMe		DIB, 2.5	56^e^
OBn	OBn	OMe	OMe				OMe		DIB, 1.9	52^j^
OBn	OBn	OBn		OMe			OMe		DIB, 1.9	57^j^
OBn	OBn	OMe	OMe				OMe	OBn	DIB, 1.9	67^j^
OBn	OMe	OMe					OMe	OBn	DIB, 1.9	61^j^
OBn	OBn	OBn		OMe			OMe	OBn	DIB, 1.9	58^j^

*^a^* Only substituents other than H are shown in the Table. Items without a reference are results reported here for the first time. *^b^* DIB = di-isobutylaluminiumhydride. *^c^* SEL = K- or L-Selectride^®^. *^d^* 2-Methyl-7-methoxyisoflavone reacted similarly in 63% yield.[21] *^e^* Ref. 21. *^f^* Ref. 24. *^g^* Ref. 16. *^h^* The corresponding tris(methoxyethoxymethoxy)flavone reacted similarly. *^i^* Ref. 23. *^j^* Ref. 22.

**Table 2:** Reduction of isoflavones to isoflavan-4-ols*^a^*

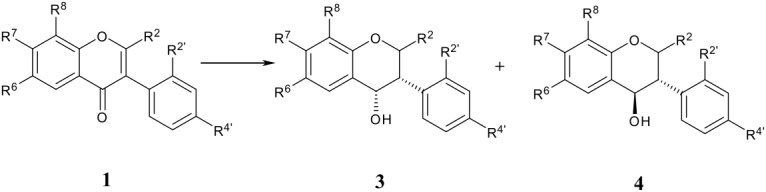										

SM	R^2^	R^2'^	R^4'^	R^6^	R^7^	R^8^	reductant, eqs, solvent	yield **3**+**4** %	**3:4** ratio	Yld **6** %

**1a**							NaBH_4_,2.5,EtOH	86	70:30	12
							LiBH_4_,2.5,THF	56	80:20	40
							NaBH_4_,2.5,MeOH	57^b^	**3** only	
							NaBH_4_,2,EtOH	75^c,d^		
							NaBH_4_,2,diglyme	88^d,e^		
							NaBH_4_,3.5,PdCl_2_,aq. THF	85	82:18	
							NaBH_4_,H_3_BO_3_,2.5, EtOH	98	71:29	
							NaBH_4_,CeCl_3_,1.0, DMSO	99	**3** only	
							NaBH_4_,AlCl_3_,excess., digl.	81^d^		
							Zn(BH_4_)_2_,4,Et_2_O	69	62:38	19
							LiEt_3_BH,4,THF	50	70:30	
							B_2_H_6_, excess,THF	76^c,d^		
**1b**					OMe		NaBH_4_,2.5,EtOH	90	70:30	9
							NaBH_4_,H_3_BO_3_,3.1, EtOH	80^f^	**3** only	
							NaBH_4_,H_3_BO_3_,2.5, EtOH	97	70:30	
							NaBH_4_,CeCl_3_,1.5, DMSO	88	**3** only	12
							LiBH_4_,10,THF	54	70:30	37
							LiEt_3_BH,4,THF	55	65:35	
**1c**			OMe		OMe		NaBH_4_,2.5,MeOH	37^b^	3 only	
							NaBH_4_,2.5,EtOH	88	70:30	10
							NaBH_4_,CeCl_3_,2.5, DMSO	86	3 only	10
							NaBH_4_,H_3_BO_3_,2.5, EtOH	96	70:30	
							LiBH_4_,10,THF	70	77:23	10
							Zn(BH_4_)_2_,2,Et_2_O	74	62:38	
							LiEt_3_BH,4,THF	36	78:22	
**1**		OMOM	OMOM		OMOM		NaBH_4_,15.9,EtOH, THF LiBH_4_,10,THF	87^g^ 82^g^	65:35 57:43	
**1**	Me			Br	OMe		NaBH_4_,4.2,EtOH	25^e,h^		
**1**	Me			Br	OBn		NaBH_4_,4.2,EtOH	22^e,h^		
**1**	Me				OMe	Br	NaBH_4_,4.2,EtOH	20^e,h^		
**1**	Me			Br	OMe	Br	NaBH_4_,4.2,EtOH	20^e,h^		

*^a^* Only substituents other than H are shown. According to our results, OH substituted isoflavones are not reduced, nor are such reactions reported in the literature. Items without a reference are results reported here for the first time. *^b^* Ref. 25. *^c^* Product was given as 2-isoflaven-4-ol. *^d^* Ref. 28. *^e^* Product ratio not given. *^f^* Ref. 26. *^g^* Ref. 16. *^h^* Ref. 27.

### Isoflavanols (3, 4)

Full reduction at the heterocyclic ring of isoflavones by LiBH_4_ or NaBH_4_ leads to isoflavanols in 20–91% yield (Table 2), [16,25–28]except with hydroxy substituted substrates which do not react at all. Apparently the first step involves a 1,4-addition to give the isoflavanone enolate which picks up a proton from the solvent and is reduced further to the saturated alcohol. There are no reports of the intermediacy of the alternative 1,2-reduction products, the allylic alcohols **11** which in fact appear very incompletely known in the chemical literature (see below). Similarly, the reduction of isoflavanones **2** by LiBH_4_, NaBH_4_, L-Selectride^®^ or Li(*t*-BuO)_3_AlH gives mixtures of *cis*-**3** and *trans*-isoflavan-4-ols **4** (Table 3). [16,29,34] Isoflavanones are reduced by electrophilic hydrides (borane-tetrahydrofuran, bis-*tert*-butylthioethane borane) diastereoselectively to *cis*-isoflavan-4-ols, but a large excess of the reducing agent is usually needed [29].

**Table 3:** Reduction of isoflavanones to isoflavan-4-ols*^a^*

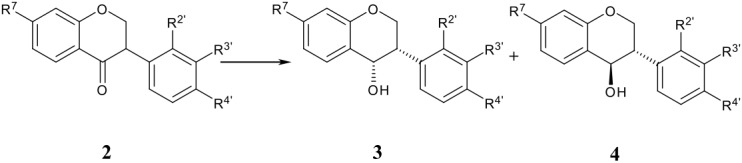							

SM	R^2'^	R^3'^	R^4'^	R^7^	reductant, eqs, solvent	yield **3+4** %	**3:4** ratio

**2a**					NaBH_4_, ng.,EtOH NaBH_4_,2,MeOH,THF Li(t-BuO)_3_AlH,10,THF B_2_H_6_,80,THF	99^b^ 99^c^ 99^c^ 98^c^	70:30 42:58 34:66 **3** only
**2b**				OMe	NaBH_4_,2,MeOH,THF NaBH_4_,1.2,EtOH Li(t-BuO)_3_AlH,10,THF L-Selectride, ng B_2_H_6_,80,THF BTED^g^,0.7,THF	99^c^ 67^d^ 99^c^ 99^c^ 99^c^ 98^c^	44:56 67:33 33:67 37:63 **3** only **3** only
**2**			OH	OH	LiBH_4_,6.9,THF	94^e^	70:30
**2**			OTBDMS	OTBDMS	LiBH_4_,2,THF	96^f^	70:30
**2c**			OMe	OMe	NaBH_4_,2,THF,MeOH NaBH_4_,1.2,EtOH Li(t-BuO)_3_AlH,10,THF B_2_H_6_,80,THF	99^c^ 50^d^ 99^c^ 97^c^	43:57 **3** only 34:66 **3** only
					BTED^g^,0.7,THF	97^c^	**3** only
**2**		OMe	OMe	OMe	NaBH_4_,2,THF,MeOH	99^c^	36:64
					Li(t-BuO)_3_AlH,10,THF	99^c^	32:68
					B_2_H_6_,80,THF	98^c^	**3** only
					BTED^g^,0.7,THF	98^c^	**3** only
**2**	OMOM		OMOM	OMOM	LiBH_4_,10,THF	73^h^	55:45
					LiAlH_4_,17,THF	78^h^	45:55
					NaBH_4_,15.9,THF,MeOH	91^h^	70:30

*^a^* Only substituents other than H are shown. ng not given *^b^* Ref. 31. *^c^* Ref. 29. *^d^* Ref. 30. *^e^* Ref. 32. *^f^* Ref. 33. *^g^* bis-*t*-butylthioethane diborane. *^h^* Ref. 16.

Although it was realized by the early workers that diastereomeric mixtures of isoflavanols would presumably be formed, there were no reliable methods to determine their structures. In some papers, the products are summarily assigned the *cis* [25–26] or *trans* [35–38] structures. However, rigorous NMR analysis has recently made it possible to establish *cis*- and *trans*-structures for the isoflavanol products and to study their conformational equilibria [32,34]. Borohydride reductions generally give a small preference for the *cis* products as suggested by Cram's rule [39]. We are not aware of any examples in the literature of single enantiomers of isoflavanone or isoflavanol metabolites, nor have such compounds been reported as hydride reduction products of isoflavones. In view of the significant biological properties of the reduced metabolites it will be interesting to examine the behaviour of the pure enantiomers.

### 2-Isoflaven-4-ols (11)

**Figure 2:**
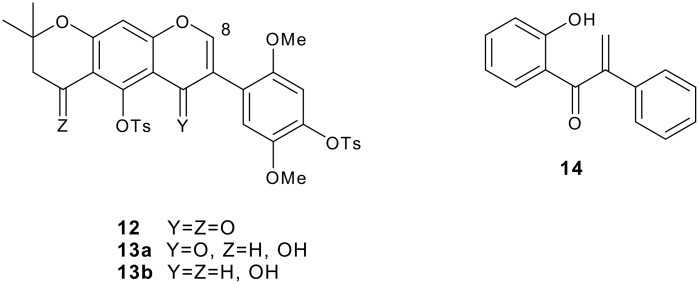
2-Isoflaven-4-ols and 1-(2-hydroxyphenyl)-2-phenyl-2-propen-1-one

There are very few reports of this class of compounds, either from reductive processes or otherwise. In 1965, the reduction of the parent isoflavone by NaBH_4_ in EtOH or diborane in THF was claimed [28] to furnish 2-isoflaven-4-ol in 75–76% yield, but unfortunately the characterization of this product relied on elemental analysis only. More recently, Japanese workers [40] reported that the reduction of **12** (Figure 2) by NaBH_4_ in the presence of PdCl_2_ in THF-H_2_O gave a 1:1 mixture of the ketone **13a** and the diol **13b** (Figure 2). A ^1^H NMR spectrum was given for the latter isoflavenol but there appear to be certain discrepancies, notably in the δ value (8.69) reported for the H-8 which is some 2 δ units in excess of what would be expected for such a vinyl ether proton. Thus more work is required to fully confirm the nature of this class of reduction products. In the event, in our studies the reduction of isoflavone **1a** by NaBH_4_ in the presence of PdCl_2_ in THF-H_2_O gave a 82:18 mixture of *cis*- and *trans*-isoflavan-4-ol **3a**, **4a** while no 2-isoflaven-4-ols were observed. The absence of such 1,2-reduction products, or structures conceivably derivable thereof such as 2- or 3-isoflavenes (**7**, **8**), even in the CeCl_3_-complexed NaBH_4_ reductions, must reflect the good stabilization obtainable via resonance heteroring stabilization (**10**) in the isoflavones. As already mentioned, this is in contrast to the behaviour of simple non-flavonoid β-alkoxy-α,β-unsaturated ketones which prefer 1,2-attack by hydride.

### Isoflavenes (7, 8) and isoflavans (9)

In the early work, [41] there is a mention of 7,4'-dimethoxy-2-methyl-3-isoflavene being obtained in 15% yield from the reduction of the corresponding isoflavone by LiAlH_4_ in Et_2_O-benzene but there is no structural data on the product other than elemental analysis, itself quite accurate. Similarly 2',4'-dimethoxy-3',6,7-trihydroxyisoflav-3-ene was reported from the reduction of 2',4'-dimethoxy-3',6,7-trihydroxyisoflavan-4-one with LiAlH_4_ in low yield [42]. More recent work by us [43] and others [16,44] would indicate that the normal course of LiAlH_4_ reduction of isoflavones leads to deoxybenzoins and propenols (see below and Table 4).

**Table 4:** Reduction of isoflavones to α-methyldeoxybenzoins and 1,2-diaryl-2-propen-1-ols*^a^*

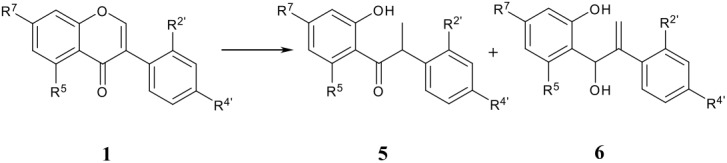						
**a** all R groups = H						
**b** R^7^ = Me, other R groups = H						
**c** R^4^ = R^7^ = Me, other R groups = H						

R^2'^	R^4'^	R^5^	R^7^	reductant, eqs, solvent	yield (%)	
					of **5**	of **6**

				LiAlH_4_, 2.5,THF	27^b^	48
				Li(t-BuO)_3_AlH, 5,THF	74	6
				Red-Al, 2.5,THF	12	50
				LiBH_4_, 10, THF	9	68
			OMe	LiAlH_4_, 2.5,THF	29^b^	50
				LiAlH_4_, 1, THF	62^c^	-
				Li(t-BuO)_3_AlH,10,THF	88	12
				LiBH_4_, 10, THF	7	37
	OMe		OMe	LiAlH_4_,3.3,THF	27^b^	70
				Li(t-BuO)_3_AlH,10,THF	88	12
				LiBH_4_, 10, THF	5	10
			OH	LiAlH_4_,3.3,THF	60^b^	-
				Li(t-BuO)_3_AlH,8,THF	-	-
	OH		OH	LiAlH_4_,5.5,THF	42^b^	-
				Li(t-BuO)_3_AlH,10,THF	-	-
	OMe		OH	LiAlH_4_,3.3,THF	42^b^	-
	OH	OH	OH	LiAlH_4_,5.5,THF	66^b^	-
	OMe	OH	OH	LiAlH_4_,4.3,THF	70^b^	-
	OH		OMe	LiAlH_4_,3.3,THF	17^b^	34
OMOM	OMOM		OMOM	LiAlH_4_,7.9,THF	6^d^	-

*^a^* Only substituents other than H are shown in the Table. Items without a reference are results reported here for the first time. *^b^* Ref. 43. *^c^* Ref. 44. *^d^* Ref. 16.

Published syntheses of 2- and 3-isoflavenes involve the reduction of 3-arylcoumarins, [45–47] the corresponding aldehyde hemiacetals, [7] isoflavylium salts [48–50] or of isoflavones by the Clemmensen reaction [51]. Low-yielding non-reductive routes to 2- and/or 3-isoflavenes have also been reported [52–53]. 2-Isoflavenes **7** however remain mostly poorly characterized, and some of the NMR spectral details reported [7,51–52] appear inconsistent with the 2-isoflavenoid structure. As far as the NMR spectra of 2-isoflavenes are concerned, a recent study clears this issue by 2D NMR work on a natural 2-isoflavene, [54] establishing that the H-2 and H-4 protons appear at δ 6.87 and 3.61, respectively, much as expected by correlation data shift calculations. Isoflavans (**9**) have not been prepared by hydride reductions, but by catalytic hydrogenation of isoflavones [10,16–20].

### Deoxybenzoins (5) and propenols (6)

Isoflavanones undergo a facile retro-Michael-type ring opening [21,55] under basic conditions to give the propenone intermediate **14** (Figure 2), sometimes considered [56] to be an independent isoflavone metabolite but presumably just an artefact in reality. As regards the synthesis of isoflavanones by DIBAH reduction of isoflavones (see above), we found that unless the workup is done with cold methanolic HCl, some amount of the propenone **14** will be formed and reduced further to the deoxybenzoin **5**. If on the other hand the deoxybenzoins are the actual synthetic targets, the reducing agent of choice is LiAlH_4_ in THF. This works very well for isoflavones bearing a hydroxy group at C-7 such as genistein, but in isoflavones lacking a 7-OH group another reaction pathway competes leading to the propenols **6** [43] as byproducts (see below). We have discussed a possible mechanism to explain these hydroxyl-dependent divergent pathways [43].

To summarize, all hydride addition reactions with isoflavones appear to involve an initial 1,4-addition to give the isoflavanone enolate. In a hydroxylic solvent, or even on workup under basic conditions, the ketone is generated, and reduced further to the saturated alcohol (NaBH_4_). In a nonprotic solvent, the β-aryloxyenolate will undergo a retro-Michael addition, giving the phenolate anion of the ring opened 2-propen-1-one which may undergo a 1,2- or 1,4-addition of hydride (LiAlH_4_, LiBH_4_). If the *O*-metal bond in the initial enolate is very tight, the ring opening does not occur and allows the isolation of the isoflavanone (DIBAH, Selectrides^®^). The presence and number of hydroxy or alkoxy substituents in the substrates does not have a major effect in these reductions except in the case of NaBH_4_ reduction which fails completely presumably due to solubility reasons, and the LiAlH_4_ reduction where the outcome depends on the presence or absence of an OH group at C-7. Based on our and previous results by other workers, the reducing agents of choice for the synthesis of reduced isoflavonoids are as follows:

isoflavanones (**2**) from non-hydroxylated isoflavones DIBAH or Selectrides^®^

isoflavanones (**2**) from hydroxylated isoflavones DIBAH

*cis*-isoflavanols (**3**) from non-hydroxylated isoflavones NaBH_4_/CeCl_3_

*cis*-isoflavanols (**3**) from hydroxylated isoflavones no good methods

*cis*-isoflavanols (**3**) from non-hydroxylated isoflavanones B_2_H_6_ or BTED

*trans*-isoflavanols (**4**) from isoflavones no good methods

*trans*-isoflavanols (**4**) from isoflavanones Li(*t*-BuO)_3_AlH

2-isoflaven-4-ols (**11**) from isoflavones uncertain

isoflavenes (**7**, **8**) from isoflavones Clemmensen

isoflavans (**9**) from isoflavones H_2_, Pd/BaSO_4_

α-methyldeoxybenzoins (**5**) from non-hydroxylated isoflavones Li(*t*-BuO)_3_AlH

α-methyldeoxybenzoins (**5**) from hydroxylated isoflavones LiAlH_4_

1,2-diaryl-2-propen-1-ols (**6**) from non-hydroxylated isoflavones LiBH_4_ or LiAlH_4_

## Conclusion

Dietary isoflavonoids in vegetables, beans, peas and other legumes are possible cancer preventing agents, particularly in hormone based cancers such as breast and prostate cancer. [57–59] Epidemiological studies have shown that they decrease the risk of colon cancer, osteoporosis, and coronary heart disease. [1–3] Significantly, health claims of soy foods, rich in isoflavonoids, have recently received FDA authorization [60].

**Figure 3:**
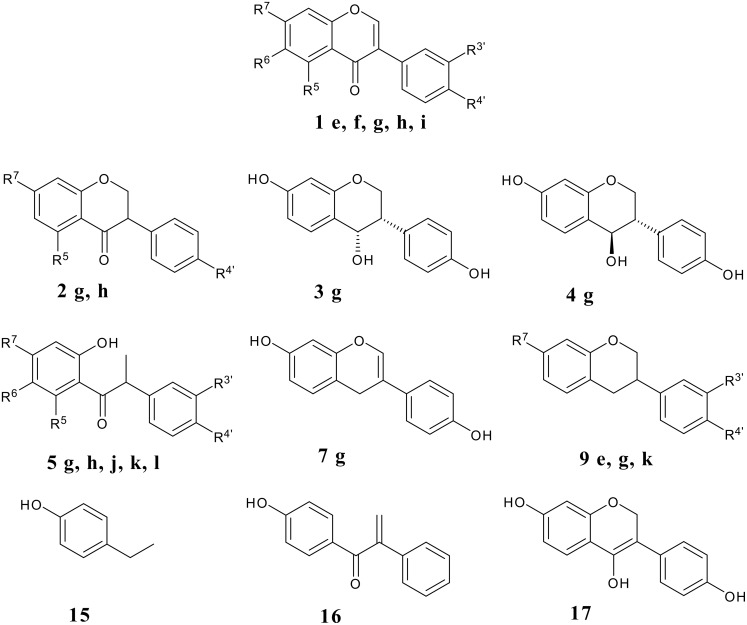
Dietary isoflavones and their metabolites in humans. **e** R^4’^=OMe, R7= OH, other R groups = H **f** R^4’^= OMe, R5=R7= OH, other R groups = H **g** R^4’^= R5=R7= OH, other R groups = H **h** R^4’^=R7= OH, other R groups = H **i** R^4’^=R7= OH, R6=OCH3, other R groups = H **j** R^4’^= R6=R7= OH, other R groups = H **k** R^3’^= R7= OH, other R groups = H **l** R^3’^=R4’=R7= OH, other R groups = H

The dietary isoflavonoids are mainly metabolized in man via reductive pathways, leading to the reduced structural types discussed above (Figure 3). These compounds are often more estrogenic than the starting isoflavones. Research interest in many fields including medicine, nutrition and biosynthesis and metabolism thus converge on the reduced isoflavonoids. The work described in this paper shows that most structural types of reduced isoflavonoids are now reliably available in satisfactory or good yields by hydride reductions. Although not discussed here, it is clear that D atoms may be introduced in the same way which is very useful in the quantitation of the naturally occurring compounds by GC-MS selected ion monitoring techniques [61].

## Supporting Information

File 1 Experimental details and characterisation data.

## References

[1] Cornwell T, Cohick W, Raskin I (2004). Phytochemistry.

[2] Cos P, De Bruyne T, Apers S, Van den Berghe D, Pieters L, Vlietinck A J (2003). Planta Med.

[3] Duncan A M, Phipps W R, Kurzer M S (2003). Best Pract Res Clin Endocrinol Metab.

[4] Bezuidenhoudt B C B, Brandt E V, Roux D G (1981). J Chem Soc, Perkin Trans 1.

[5] Jain A C, Mehta A (1986). J Chem Soc, Perkin Trans 1.

[6] Shih T L, Wyvratt M J, Mrozik H (1987). J Org Chem.

[7] Liepa A J (1984). Aust J Chem.

[8] Gopal D, Rajagopalan K (1987). Indian J Chem.

[9] Szabó V, Antal E (1973). Tetrahedron Lett.

[10] Szabó V, Antal E (1976). Acta Chim Acad Sci Hung.

[11] Jensen N P, Brown R D, Schmitt S M, Windholz T B, Patchett A A (1972). J Org Chem.

[12] Gannon W F, House H O (1960). Org Synth.

[13] Danishefsky S, Kerwin J F, Kobayashi S (1982). J Am Chem Soc.

[14] Kende A S, Benechie M, Curran D P, Fludzinski P, Swenson W, Clardy J (1979). Tetrahedron Lett.

[15] Denmark S E, Habermas K L, Hite G A (1988). Helv Chim Acta.

[16] Süsse M, Johne S, Hesse M (1992). Helv Chim Acta.

[17] Adlercreutz H, Musey P I, Fotsis T, Bannwart C, Wähälä K, Mäkelä T, Brunow G, Hase T (1986). Clin Chim Acta.

[18] Luk K-C, Stern L, Weigele M (1983). J Nat Prod.

[19] Antus S, Gottsegen Á, Kolonits P, Nógrádi M (1986). Liebigs Ann Chem.

[20] Wähälä K, Valo T, Brunow G, Hase T (1989). Finn Chem Lett.

[21] Antus S, Gottsegen Á, Nógrádi M (1981). Synthesis.

[22] Antus S, Gottsegen Á, Kolonits P, Nagy Z, Nógrádi M, Vermes B (1982). J Chem Soc, Perkin Trans 1.

[23] Májor Á, Nógrádi M, Vermes B, Kajtár-Peredy M (1988). Liebigs Ann Chem.

[24] Jain A C, Kumar A, Sharma N K (1991). Indian J Chem.

[25] Yamaguchi S, Ito S, Nakamura A, Inoue N (1965). Bull Chem Soc Jpn.

[26] Anjaneylu A S R, Sri Krishna C, Ramachandra Row L (1965). Tetrahedron.

[27] Badran M M, El-Saba H M (1991). Egypt J Pharm Sci.

[28] Thakar G P, Janaki N, Subba Rao B C (1965). Indian J Chem.

[29] Chidiak H, Kirkiacharian S (1996). Arm Khim Zh.

[30] Inoue N (1964). Bull Chem Soc Jpn.

[31] Szabó V, Borbély J, Antal E (1979). Acta Chim Acad Sci Hung.

[32] Wähälä K, Koskimies J K, Mesilaakso M, Salakka A K, Leino T K, Adlercreutz H (1997). J Org Chem.

[33] Wähälä K, Salakka A, Adlercreutz H (1998). Proc Soc Exp Biol Med.

[34] Pihlaja K, Tähtinen P, Klika K D, Jokela T, Salakka A, Wähälä K (2003). J Org Chem.

[35] Anjaneyulu A S R, Rao M G, Row L R, Krishna C S (1966). Tetrahedron Lett.

[36] Anjaneyulu A S R, Krishna C S, Row L R (1965). Bull Natl Inst Sci India.

[37] Inoue N, Yamaguchi S, Fujiwara S (1964). Bull Chem Soc Jpn.

[38] Yamaguchi S, Ito S, Suzuki I, Inoue N (1968). Bull Chem Soc Jpn.

[39] Gomis M, Kirkiacharian B S (1990). Tetrahedron.

[40] Tsukayama M, Kawamura Y, Tamaki H, Kubo T, Horie T (1989). Bull Chem Soc Jpn.

[41] Bradbury R B, White D E (1953). J Chem Soc.

[42] Shoukry M M, Darwish N A, Morsi M A (1982). Gazz Chim Ital.

[43] Salakka A, Wähälä K (1999). J Chem Soc, Perkin Trans 1.

[44] Vermes B, Antus S, Gottsegen Á, Nógrádi M (1983). Liebigs Ann Chem.

[45] Bulut M (1991). Chim Acta Turc.

[46] Grese T A, Pennington L D (1995). Tetrahedron Lett.

[47] Verma P, Singh S, Dikshit D K, Ray S (1988). Synthesis.

[48] Liepa A J (1981). Aust J Chem.

[49] Bouvier P, Adrieux J, Cunha H, Molho D (1977). Bull Soc Chim Fr.

[50] Deschamps-Vallet C, Ilotse J-B, Meyer-Dayan M (1983). Tetrahedron Lett.

[51] Dudley K H, Miller H W, Corley R C, Wall M E (1967). J Org Chem.

[52] Diaz P, Gendre F, Stella L, Charpentier B (1998). Tetrahedron.

[53] Baranton F, Fontaine G, Maitte P (1968). Bull Soc Chim Fr.

[54] Miyase T, Sano M, Yoshino K, Nonaka K (1999). Phytochemistry.

[55] Szabó S, Antal E (1976). Magy Kem Foly.

[56] Kelly G E, Nelson C, Waring M A, Joannou G E, Reeder A Y (1993). Clin Chim Acta.

[57] Pollard M, Wolter W (2000). Prostate.

[58] Lamartiniere C A (2000). Am J Clin Nutr.

[59] Wiseman H (2000). Expert Opin Invest Drugs.

[R60] 60Federal register 64FR57699, U.S. Food and Drug Administration, Oct 26, 1999

[61] Adlercreutz H, Fotsis T, Lampe J, Wähälä K, Mäkelä T, Brunow G, Hase T (1993). Scand J Clin Lab Invest.

[62] Gensler W J, Johnson F, Sloan A D B (1960). J Am Chem Soc.

[63] Fisher G B, Harrison J, Fuller J C, Goralski C T, Singaram B (1992). Tetrahedron Lett.

[64] Olah G A, Wang Q, Prakash G K S (1992). Synlett.

[65] Ibrahim A-R, Abul-Hajj Y J (1990). J Nat Prod.

[66] Osawa K, Yasuda H, Maruyama T, Morita H, Takeya K, Itokawa H (1992). Chem Pharm Bull.

