# Supplementary material for: Multiple hydride reduction pathways in isoflavonoids
Source: Beilstein J Org Chem. 2006 Aug 25;2:16. doi: 10.1186/1860-5397-2-16 (PMC1630637; doi:10.1186/1860-5397-2-16)
Supplement: File 1 — Experimental details and characterisation data. [file Beilstein_J_Org_Chem-02-16-s001.doc]

**Additional Information**

**Experimental**

Melting points were determined in open capillary tubes and are uncorrected. IR spectra were recorded from KBr disks and UV spectra were recorded in 94% ethanol solution. ^1^H NMR spectra were recorded on a 200, 300 or 500 MHz and ^13^C NMR spectra on a 50, 75 or 125 MHz FT NMR spectrometers (chemical shifts in ppm). LR and HR mass spectra were obtained at 70 eV. TLC was conducted on silica gel plates and silica gel (0.040–0.063 mm, 230–400 mesh) was used for flash chromatography. Tetrahydrofuran (THF) and dimethyl sulfoxide (DMSO) were dried by distilling over CaH2 before use.

**General reduction procedures**

**Reductions with NaBH4 and H3BO3 or CeCl3•7H2O.** A solution of the isoflavone (1 eq.) in 94% EtOH (or in the presence of boric acid (1 eq.) in 94% EtOH, or of cerium chloride heptahydrate (1 eq.) in MeOH or in dry DMSO) was treated with sodium borohydride (0.5–5 eqs) in small portions with stirring at 25 °C. The reaction mixture was neutralized after 5 h–5 d with saturated NH_4_Cl solution, some water added and extracted with ether or with EtOAc. The organic phase was separated, washed with water, dried over Na_2_SO_4_ and evaporated to give a crude product, purified by flash chromatography.

**Reductions with NaBH4 and PdCl2.** Sodium borohydride was added carefully to a solution of isoflavone (1 eq.) and PdCl2 (1.4 eq.) in THF–water (4:1) with stirring at 15 °C. After 5 h at room temperature the reaction mixture was filtered and THF evaporated. The residue was poured to water and extracted with CHCl3, the extract washed with 5% aqueous NaHCO3 and the solvent evaporated to give the crude product, purified by flash chromatography.

**Reduction with LiAlH4 or LiBH4.** A solution of isoflavone (1 eq.) in dry THF was added to a solution of lithium aluminium hydride (2.5–10 eqs) in dry THF at the reaction temperature or to a solution of lithium borohydride in dry THF at –30 to +40 °C with stirring under argon. The reaction was quenched with saturated NH_4_Cl solution as described above.

**Reduction with LiAl[OC(CH3)3]3H.** A 1M solution of lithium tri-*tert*-butoxyaluminium hydride in THF (5–10 eqs) was added dropwise to a solution of the isoflavone (1 eq) in dry THF in an ice bath under argon. The reaction temperature was 25–35 °C. Reaction was quenched with saturated NH_4_Cl solution as described above.

**Reduction with DIBAH or L-Selectride® or K-Selectride®.** 1.5 M solution of di-isobutylaluminium hydride (4–25 eqs) in toluene (or a 1 M solution of lithium or potassium tri-*sec*-butylborohydride (4–9 eq.) in THF) was added under argon with stirring to a solution of isoflavone (1 eq) in dry THF at –72 °C. The reaction was quenched by adding carefully the same volume of MeOH to the cold reaction mixture which was then neutralized with 2 M HCl, poured to water and extracted with ether or EtOAc. The mixture must not be allowed to warm under these manipulations. The extract was dried with Na2SO4 and solvent evaporated to give the crude product, purified by flash chromatography.

**Reduction with Zn(BH_4_)_2_.** Zinc borohydride was prepared from ZnCl_2_ and sodium borohydride.62 Excess zinc borohydride in ether was added to the solution of isoflavone (1 eq) dissolved in the minimum amount of abs. ether and some dry THF with stirring. After 11 d the mixture was neutralized with saturated NH_4_Cl and extracted with ether. The extract was dried with Na_2_SO_4_ and solvent evaporated to give the crude product.

**Reduction with LiEt_3_BH** **.** A 1M solution of lithium triethylborohydride (4–9 eq.) in THF was added dropwise to the solution of isoflavone (1 eq) in dry THF at –20 °C under argon. After the reaction at –20 °C or at 20 °C it was quenched with water and neutralized with 2 M HCl. The mixture was extracted with CH_2_Cl_2_ or EtOAc, organic phase washed with water, dried over Na_2_SO_4_ and the solvent evaporated.

**Reduction with LiPyrrBH_3_.** LiPyrrBH_3_ was prepared from pyrrolidine, BH_3_•THF and *n*-BuLi according literature.63 Isoflavone (1 eq) was dissolved in dry THF under argon and LiPyrrBH_3_ (ccalq=0.69 M, 3 eqs) was added dropwise at room temperature. The reaction was quenched after five hours by pouring to ice water, neutralized with 2 M HCl and extracted with CH_2_Cl2 Organic phase was washed with water, dried over Na_2_SO_4_ and the solvent evaporated.

**Reduction with Et_3_SiH/NH_4_F/CF_3_COOH.**64 CF_3_COOH (10 eqs) was added during 10 min at 2 °C to a stirred solution of isoflavone (1 eq), NH_4_F (2.6 eqs) and triethylsilane (2.6 eqs) in CH_2_Cl_2_. The reaction mixture was stirred for 30 min at 2 °C and brought to room temperature. After 7 days reaction time it was quenched with ice water, extracted with CH_2_Cl_2_, and the extract dried with Na_2_SO_4_. The crude product was analyzed after the evaporation of solvent.

**Reduction with diborane.** A 1 M solution of BH_3_•THF (7.5 eqs) was added to a solution of isoflavone (1 eq) in dry THF at room temperature under argon. After 7 days reaction was quenched by adding water and oxidized by 20% H_2_O_2_ and 1 M NaOH. The mixture was extracted with CH_2_Cl_2_ and the extract dried over Na_2_SO_4_. The solvent was evaporated to give the crude product.

**Reduction with Red-Al® .** A solution of sodium bis(2-methoxyethoxy)aluminium hydride (1.5–3.3 eqs) in toluene was further dissolved in dry THF and added to the solution of isoflavone (1 eq) in dry THF at –20 to +20 °C under argon. The reaction mixture was neutralized after 2.5 h–4 d with saturated NH_4_Cl solution, some water was added and extracted with CH_2_Cl_2_. The organic phase was separated, dried over Na_2_SO_4_ and the solvent evaporated to give the crude product.

**Reduction with Bu_3_SnH.** Tributyltin hydride (2 eq.) was added dropwise to the solution of isoflavone (1 eq), Pd(PPh_3_)_4_ (0.01 eqs) and ZnCl_2_ (1 eq) at room temperature under argon (or azobisisobutyronitrile (AIBN) (0.2 eqs) in refluxing THF). After 24 h or 4 h the mixture was poured into water, extracted with CH_2_Cl_2_, dried with Na_2_SO_4_ and the crude product analyzed after the evaporation of solvent. See additional information for full experimental data.

**2,3-dihydro-3-phenyl-4*H*-1-benzopyran-4-one** (Isoflavan-4-one, **2a)** White crystals mp 76-77 °C (from Et_2_O) (lit.^65^ 75–76 °C). ^1^H-NMR (CDCl_3_), IR and UV as reported.^65^ ^13^C-NMR (CDCl_3_): δ 52.8, 72.0, 118.4, 121.6, 122.2, 128.3, 128.3, 129.2, 129.4, 135.6, 136.6, 162.2 and 192.7. m/z 224 (73%, M^+^), 165 (3) 147 (5), 121 (11), 120 (100), 104 (35) and 92 (17). HRMS calcd for C_15_H_12_O_2_, 224.0837; found, 224.0845.

**2,3-dihydro-7-methoxy-3-phenyl-4*H*-1-benzopyran-4-one** (7-Methoxyisoflavan-4-one, **2b**)  White crystals mp 89 °C (from *n*-hexane-Et_2_O) (lit.5 91–92 °C). ^1^H-NMR (CDCl_3_), IR and UV as reported;^5^ ^13^C-NMR (d_6_-acetone): δ 52.5, 56.2, 72.5, 101.5, 110.9, 115.8, 128.2, 129.4, 129.6, 129.7, 137.3, 164.5, 167.0 and 190.9. m/z 254 (92%, M^+^), 252 (34), 251 (37), 148 (23), 147 (100), 119 (32), 104 (13), 101 (20), 76 (7) and 55 (8). HRMS calcd for C_16_H_14_O_3_, 254.0942; found, 254.0952.

**2,3-dihydro-7-methoxy-3(4-methoxyphenyl)-4*H*-1-benzopyran-4-one** (4’,7-Dimethoxyisoflavan-4-one, **2c**) White crystals mp 129 °C (from Et_2_O) (lit.^5^ 134–135 °C). ^1^H-NMR (CDCl_3_), IR and UV as reported.^5^ ^13^C-NMR (CDCl_3_): δ 51.1, 55.2, 55.6, 71.9, 100.5, 110.1, 114.2, 114.8, 127.2, 129.4, 129.5, 158.9, 163.4, 166.1 and 191.2. m/z 284 (22%, M^+^), 282 (10), 150 (3), 135 (10), 134 (100), 119 (8), 91 (6) and 58 (2). HRMS calcd for C_17_H_16_O_4_, 284.1049; found, 284.1058.

**2,3-dihydro-7-hydroxy-3-phenyl-4*H*-1-benzopyran-4-one** (7-Hydroxyisoflavan-4-one, **2d**) Pale yellow crystals mp 175 °C (from EtOH) (lit.5 174–175 °C). ^1^H-NMR (acetone-d_6_), IR and UV as reported.^5^ ^13^C-NMR (acetone-d_6_): δ 52.6, 72.5, 103.5, 111.5, 115.4, 128.1, 129.4, 129.6, 130.2, 137.4, 164.5, 165.2 and 190.7. m/z 240 (70%, M^+^), 137 (13), 136 (100), 108 (15), 104 (33) and 58 (18). HRMS calcd for C_15_H_12_O_3_, 240.0787; found, 240.0796.

**2,3-dihydro-7-hydroxy-3(4-methoxyphenyl)-4*H*-1-benzo­pyran-4-one** (7-Hydroxy-4’-methoxyisoflavan-4-one, **2e**) Pale brown crystals mp 195 °C (from EtOH) (lit.5 184–186 °C). ^1^H-NMR (acetone-d_6_), IR and UV as reported.^5^ ^13^C-NMR (acetone-d_6_): δ 51.6, 55,3, 72.4, 103.3, 111.2, 114.6, 115.2, 129.0, 130.1, 130.4, 159.8, 164.3, 165.0 and 190.9. m/z 270 (26%, M^+^), 252 (3), 135 (14), 134 (100), 119 (10), 91 (6) and 58 (10). HRMS calcd for C_16_H_14_O_4_, 270.0892; found, 270.0901.

**2,3-dihydro-5,7-dihydroxy-3-(4-methoxyphenyl)-4*H*-1-benzopyran-4-one** (5,7-Dihydroxy-4’-methoxyisoflavan-4-one, **2f**) white crystals mp 173–174 °C (from EtOH) (lit.^66^ 175–177 °C), ^1^H-NMR (acetone-d_6_), ^13^C-NMR (acetone-d_6_), IR and UV as reported.^66^ HRMS calcd for C_16_H_14_O_5_, 286.0842; found, 286.0846.

**cis-2,3-dihydro-3-phenyl-4*H*-1-benzopyran-4-ol** (*cis*-Isoflavan-4-ol, **3a**) White crystals mp 76 °C (from *n*-hexane–Et_2_O) (lit.29 75 °C); UV (94% EtOH) λmax nm (ε): 204 (34200), 216sh (18400) and 283 (3300), ^1^H-NMR (CDCl_3_): δ 3.35 (dt, *J* = 11.7, 2.9 Hz, 1H), 4.31 (ddd, *J* = 10.6, 2.9, 1.1 Hz, 1H), 4.58 (dd, *J* = 11.7, 10.6 Hz, 1H), 4.75 (br s, 1H), 6.88–6.97 (m, 2H) and 7.20–7.37 (m, Ar H, 7H); ^13^C-NMR (CDCl_3_): δ 44.2, 64.0, 67.1, 116.9, 120.6, 123.6, 127.4, 128.4, 128.8, 129.9, 130.6, 137.7 and 154.2. m/z 226 (16%, M^+^), 222 (13), 209 (13), 208 (86), 207 (100), 194 (7), 178 (19), 165 (8), 131 (27), 122 (30) and 104 (33). HRMS calcd for C_15_H_14_O_2_, 226.0994; found, 226.0999.

***trans*-2,3-dihydro-3-phenyl-4*H*-1-benzopyran-4-ol** (*trans*-Isoflavan-4-ol, **4a**) White crystals mp 97 °C (from *n*-hexane–Et_2_O) (lit.29 98 °C); UV (94% EtOH) λmax nm (ε): 202 (47200) and 276 (300); ^1^H-NMR (CDCl_3_): δ 3.19 (td, *J* = 8.3, 7.3, 3.9 Hz, 1H), 4.27 (dd, *J* = 11.2, 8.3 Hz, 1H), 4.38 (dd, *J* = 11.2, 3.9 Hz, 1H), 4.99 (m, 1H), 6.87–6.99 (m, 2H) and 7.20–7.52 (m, Ar H, 7H); ^13^C-NMR (CDCl_3_): δ 47.1, 68.1, 69.4, 116.5, 120.9, 124.7, 127.6, 128.0, 128.1, 129.0, 129.3, 138.6 and 154.2. m/z 226 (8%, M^+^), 222 (17), 209 (14), 208 (83), 207 (100), 194 (11), 178 (21), 165 (12), 131 (26) and 122 (15). HRMS calcd for C_15_H_14_O_2_, 226.0994; found, 226.0983.

***cis*-2,3-dihydro-7-methoxy-3-phenyl-4*H*-1-benzopyran-4-ol** (*cis*-7-Methoxyisoflavan-4-ol, **3b**) White crystals mp 142 °C (from Et_2_O) (lit.29 144 °C); UV (94% EtOH) λmax nm (ε): 207 (45700) and 278 (3830). ^1^H-NMR (CDCl_3_): δ 3.32 (dt, *J* = 11.7, 2.9 Hz, 1H), 3.79 (s, OCH_3_, 3H), 4.34 (ddd, *J* = 10.6, 2.9, 1.1 Hz, 1H), 4.62 (dd, *J* = 11.7, 10.6 Hz, 1H), 4.79 (br s, 1H), 6.45 (d, *J* = 2.2 Hz, 1H), 6.54 (dd, *J* = 8.4, 2.2 Hz, 1H) and 7.20–7.40 (m, Ar H, 6H). ^13^C-NMR (CDCl_3_): δ 44.6, 55.6, 64.3, 67.0, 101.5, 108.2, 116.5, 128.1, 128.6, 129.1, 131.5, 138.0, 155.5 and 161.3. m/z 256 (13%, M^+^), 252 (75), 239 (18), 238 (100), 237 (95), 224 (14), 209 (30), 161 (32), 152 (53) and 58 (42). HRMS calcd for C_16_H_16_O_3_, 256.1099; found, 256.1108.

***trans*-2,3-dihydro-7-methoxy-3-phenyl-4*H*-1-benzopyran-4-ol** (*trans*-7-Methoxyisoflavan-4-ol, **4b**) White crystals 130 °C (from Et_2_O) (lit.29 135 °C); UV (94% EtOH) λmax nm (ε): 205 (56600) and 280 (3780). ^1^H-NMR (CDCl_3_): δ 3.16 (td, *J* = 8.3, 7.3, 3.9 Hz, 1H), 3.79 (s, 7-OCH_3_, 3H), 4.28 (dd, *J* = 11.3, 8.3 Hz, 1H), 4.39 (dd, *J* = 11.3, 3.9 Hz, 1H), 4.93 (dd, 7.3, 5.4Hz 1H), 6.41 (d, *J* = 2.4 Hz, 1H), 6.56 (dd, *J* = 8.8, 2.4 Hz, 1H), and 7.23–7.37 (m, Ar H, 6H); ^13^C-NMR (CDCl_3_): δ 47.2, 55.4, 68.1, 69.1, 101.1, 108.1, 117.0, 127.5, 128.0, 128.9, 129.3, 138.8, 155.4 and 160.7. m/z 256 (6%, M^+^), 252 (58), 239 (17), 238 (100), 237 (95), 209 (23), 165 (30), 161 (30), 152 (27) and 58 (21). HRMS calcd for C_16_H_16_O_2_, 256.1099; found, 256.1098.

***cis*-2,3-dihydro-7-methoxy-3-(4-methoxyphenyl)-4*H*-1-benzo­pyran-4-ol** (*cis*-4’,7-Dimethoxyisoflavan-4-ol, **3c**) White crystals mp 145 °C (from Et_2_O) (lit.29 144 °C); UV (94% EtOH) λmax nm (ε): 207 (60900), 226sh (35000) and 276 (7170). ^1^H-NMR (CDCl_3_): δ 3.28 (dt, *J* = 11.7, 2.9 Hz, 1H), 3.79 (s, OCH_3_, 3H,), 3.82 (s, OCH_3_, 3H), 4.31 (ddd, *J* = 10.6, 2.9, 1.1 Hz, 1H), 4.57 (dd, *J* = 11.7, 10.6 Hz, 1H), 4.75 (br s, 1H), 6.44 (d, *J* = 2.2 Hz, 1H), 6.55 (dd, *J* = 2.2, 8.4 Hz, 1H), 6.93 (d, *J* = 8.4 Hz, 2H) and 7.18–7.28 (m, 3H); ^13^C-NMR (CDCl_3_): δ 43.5, 55.3, 64.4, 66.8, 101.2, 107.9, 114.3, 116.3, 129.4, 129.7, 131.3, 155.3, 158.9 and 161.0. m/z 286 (5%, M^+^), 283 (20), 282 (100), 268 (80), 267 (95), 254 (22), 239 (29), 161 (10), 135 (10) and 134 (33). HRMS calcd for C_17_H_18_O_4_, 286.1205; found, 286.1209.

***trans-*2,3-dihydro-7-methoxy-3-(4-methoxyphenyl)-4*H*-1-benzopyran-4-ol** (*trans*-4’,7-Dimethoxyisoflavan-4-ol, **4c**) White crystals mp 125 °C (from Et_2_O) (lit.29 123 °C) UV (94% EtOH) λmax nm (ε): 205 (59200), 225sh (23500) and 277 (4970); ^1^H-NMR (CDCl_3_): δ 3.09 (td, *J* = 8.3, 7.3, 3.9 Hz,1H), 3.79 (s, OCH_3_, 6H), 4.17-4.39 (m, 2H), 4.86 (dd, *J* = 7.3, 5.4 Hz, 1H), 6.40 (d, *J* = 2.4 Hz, 1H), 6.56 (dd, *J* = 8.8, 2.4 Hz, 1H), 6.87 (d, *J* = 8.8 Hz, 2H), 7.15 (d, *J* = 8.8 Hz, 2H) and 7.35 (d, *J* = 8.8 Hz, 1H); ^13^C-NMR (CDCl_3_): δ 46.3, 55.3, 68.2, 69.2, 101.1, 108.0, 114.4, 117.1, 129.0, 129.4, 130.7, 155.4, 159.0 and 160.6. m/z 286 (3%, M^+^), 282 (27), 269 (17), 268 (100), 267 (95), 253 (14), 237 (8), 161 (10), 152 (4) and 134 (32). HRMS calcd for C_17_H_18_O_4_, 286.1205; found, 286.1201.
